# Strengthening Clinical Governance and Public Health Interventions to Improve Drug-Resistant Tuberculosis Outcomes in Rural South Africa

**DOI:** 10.3390/healthcare13172093

**Published:** 2025-08-22

**Authors:** Mojisola Clara Hosu, Urgent Tsuro, Ntandazo Dlatu, Lindiwe Modest Faye, Teke Apalata

**Affiliations:** 1 Department of Laboratory Medicine and Pathology, Faculty of Medicine and Health Sciences, Walter Sisulu University, Mthatha 5117, South Africa; tsurourgent@gmail.com (U.T.); lfaye@wsu.ac.za (L.M.F.); tapalata@wsu.ac.za (T.A.); 2 Department of Public Health, Faculty of Medicine and Health Sciences, Walter Sisulu University, Mthatha 5117, South Africa; ndlatu@wsu.ac.za

**Keywords:** drug-resistant tuberculosis, clinical governance, public health interventions, treatment outcomes, survival analysis, rural Eastern Cape

## Abstract

**Background/Objectives:** Drug-resistant tuberculosis (DR-TB) presents significant challenges to public health, particularly in rural South Africa, where limited infrastructure, high HIV co-infection rates, and weak clinical governance contribute to poor treatment outcomes. This study evaluates treatment trajectories and the impact of clinical governance and public health interventions on DR-TB outcomes in the rural Eastern Cape. **Methods**: A retrospective cohort study was conducted among 323 laboratory-confirmed DR-TB patients treated between 2018 and 2021. Kaplan–Meier curves and Cox proportional hazards analysis identified predictors of unfavorable outcomes. Logistic regression analysis simulated the impact of enhanced clinical governance scenarios on treatment success. **Results**: Treatment outcomes included cure (36.2%), completion (26.0%), loss to follow up (LTFU) (9.0%), death (9.3%), failure (2.2%), and transfer (9.3%). The median treatment duration was 10 months (IQR: 9–11). Survival analysis indicates the highest risk of death and LTFU occurred in the first 6–8 months of treatment. Multivariate Cox regression revealed that primary (HR = 0.39; 95% CI: 0.23–0.68; *p* = 0.0017) and secondary education (HR = 0.50; 95% CI: 0.31–0.85; *p* = 0.0103) were significantly protective. Paradoxically, patients with pre-XDR (HR = 0.13; *p* = 0.034) and XDR TB (HR = 0.16; *p* = 0.043) showed lower hazard of poor outcomes, likely due to early mortality or referral. HIV-negative status was associated with higher risk of poor outcomes (HR = 1.74; *p* = 0.010). Simulations suggested that improved clinical governance via better follow-up, TB/HIV integration, and adherence support could improve treatment success by up to 20 percentage points in high-impact scenarios. **Conclusions**: Strengthening clinical governance through targeted interventions could substantially reduce LTFU and mortality, especially in vulnerable subgroups. A coordinated, patient-centered approach is critical for improving DR-TB outcomes in rural, high-burden settings.

## 1. Introduction

Drug-resistant tuberculosis (DR-TB) remains one of the most complex challenges facing global TB control efforts, particularly in low-resource, high-burden settings. South Africa continues to be one of the countries with the highest burden of both multidrug-resistant TB (MDR-TB) and extensively drug-resistant TB (XDR-TB), accounting for a significant proportion of global DR-TB cases [1]. Within South Africa, the rural Eastern Cape province is disproportionately affected due to its high levels of poverty, limited healthcare infrastructure, and co-existing HIV epidemic [2], similar to other African countries [3,4].

While pharmacological management of DR-TB has improved over the past decade with the introduction of shorter regimens and new drugs like bedaquiline and delamanid, treatment outcomes remain suboptimal. Data indicates that cure and treatment completion rates for DR-TB are still well below global targets, with high rates of loss to follow-up (LTFU), mortality, and treatment failure, especially in rural settings [5,6]. These poor outcomes are often driven not just by biological or pharmacological factors, but by systemic weaknesses in clinical governance and public health response such as fragmented TB/HIV integration, inconsistent patient follow-up, and insufficient community-level engagement [7].

Clinical governance is the framework through which healthcare organizations are accountable for continually improving the quality of their services [8,9]. Clinical and care governance, while ensuring accountability in quality of health services rendered, also enhances better social care [10], thus, playing a critical role in ensuring the effectiveness of DR-TB programs. In the context of rural South Africa, the implementation of robust governance structures is often hindered by workforce shortages, limited training, and data management challenges. Similarly, public health interventions such as community-based care models, digital adherence technologies, and psychosocial support systems remain underutilized despite their proven efficacy in improving treatment adherence and retention [11,12,13,14].

Survival analysis provides a valuable methodological lens for understanding the temporal dynamics of DR-TB treatment, including when adverse events such as death or LTFU are most likely to occur. These insights can inform clinical audits, trigger early interventions, and strengthen public health planning at both facility and policy levels. This study aims to apply survival analysis techniques to a cohort of DR-TB patients in rural Eastern Cape, with the dual objectives of identifying high-risk periods and population subgroups and informing improvements in clinical governance and public health strategies.

## 2. Materials and Methods

### 2.1. Study Design and Setting

This study employed a retrospective cohort design to investigate treatment outcomes and associated risk factors among patients diagnosed with DR-TB in the rural Eastern Cape province of South Africa. The region is characterized by a high burden of both TB and HIV, limited healthcare infrastructure, and socioeconomic deprivation.

### 2.2. Study Population

The cohort consisted of 323 patients diagnosed with laboratory-confirmed DR-TB, with treatment initiated between February 2018 and December 2021. Patients were enrolled from multiple rural healthcare facilities offering decentralized DR-TB care in the Eastern Cape.

### 2.3. Inclusion and Exclusion Criteria

The study included patients who had confirmed diagnosis of DR-TB, initiated treatment during the study period, and had complete clinical records with adequate follow-up data. Patients were excluded if they had missing or incomplete key clinical variables such as HIV status, previous treatment history when either new or relapse patients, body mass index (BMI) category, or treatment outcomes, or if they were transferred into the study facilities without sufficient treatment history to assess outcomes accurately.

### 2.4. Data Collection

Patient demographic, clinical, and treatment outcome data were extracted from facility TB registers and medical records using a standardized data abstraction tool. Variables collected included age, sex, BMI, TB category, HIV status, comorbidities, drug resistance type, previous TB treatment history, and final treatment outcome.

BMI is considered the clinical standard for assessing overall adiposity in adults, calculated using clinic-grade digital scales and stadiometers for height. BMI was calculated as weight in kilogram (kg) divided by height in square meters (kg/m^2^). Body weight was measured to the nearest 0.01 kg in the standing position using a Soehnle scale (Soehnle-Waagen Gmbh Co., Muurhardt, Germany), and height was measured to the nearest 0.1 m by stadiometer in standing position with closed feet, without shoes. BMI was categorized in accordance with WHO [15] as underweight (<18.5 kg/m^−2^), normal (18.5–24.9 kg/m^2^), overweight (25.0–29.9 kg/m^2^), and obese (>30.0 kg/m^2^). The Diagnostic and Statistical Manual of Mental Disorders (DSM-5) Text Revision (DSM-5-TR) criteria was the screening standard for mental illness across various subjects, which provided descriptions, symptoms, and other criteria for diagnosing mental disorders. Additionally, the International Classification of Diseases (ICD), developed by the WHO, was used [16,17].

### 2.5. Outcome Measures

The primary outcome was time to an unfavorable treatment outcome, including LTFU, treatment failure, death, or transfer out. Favorable outcomes were defined as cure or treatment completion according to WHO definitions. Patients still on treatment at the end of the follow-up period were censored.

### 2.6. Statistical Analysis

Descriptive statistics were used to present baseline characteristics and treatment outcomes. The variables were assessed for multicollinearity using a pairwise Pearson correlation matrix. A correlation of >0.8 the presence of multicollinearity. The events per variable (*EPV*) were calculated using the following formula:

$$EPV=\frac{Number\;ofEvents\;}{Number\;of\;Predictor\;variables}$$

Kaplan–Meier survival curves were generated to estimate time-to-event distributions, and log-rank tests assessed differences across subgroups. Cox proportional hazards regression was used to identify predictors of unfavorable outcomes, with variables significant at *p* < 0.1 in univariate analysis included in the multivariate model. Statistical significance was set at *p* < 0.05. Analyses were performed using Python version 3.8. and R version 4.1.1 software, the survival package in R was used for survival analysis. A *p* < 0.05 was considered significant.

## 3. Results

### 3.1. Baseline Characteristics

Among the 323 patients included in the study, 56% were male and the median age was 33 years (IQR: 26–42). Education levels were low, with 20% having no formal education and only 10% having tertiary education. Most participants had no income (83%) and were classified as minors or unemployed (Table 1). The majority (62%) were HIV-positive. Most patients had pulmonary TB (99%), with MDR-TB (43%) and RR-TB (45%) being the most common resistance patterns. (Table 2).

### 3.2. Treatment Outcomes

Treatment outcomes were as follows: cure (36.2%), treatment completion (26.0%), LTFU (9.0%), treatment failure (2.2%), death (9.3%), transfer out (9.3%), and still on treatment at study close (8.1%). The median treatment duration was 10 months (IQR: 9–11).

Clinical characteristics in Table 2 significantly influenced treatment outcomes among DR-TB patients. Normal BMI was associated with higher cure and completion rates, while underweight patients faced increased mortality and LTFU. New TB cases had the best outcomes, whereas those with prior treatment especially PT2 were more likely to fail treatment. HIV co-infection was common and linked to higher rates of death and failure, while HIV-negative patients had better outcomes. Most patients had no comorbidities, but those with conditions like diabetes tended to remain on treatment longer. Polyresistant and MDR-TB were more frequent and associated with worse outcomes. The median treatment duration was 10 months, varying by outcome. HIV status, treatment history, BMI, and resistance type were key predictors of treatment success. The median treatment duration was 10 months (IQR: 9–11 months), with those who completed treatment averaging 11 months (IQR: 10–12), while LTFU cases had a shorter median duration of 9 months (IQR: 6–10). Transferred-out patients had the shortest treatment period (median 2 months, IQR: 1–5), suggesting early discontinuation or referral. These findings highlight the influence of clinical and demographic factors—particularly prior treatment history, HIV status, BMI, and drug resistance type—on treatment outcomes and duration in DR-TB patients.

**Figure 1:**
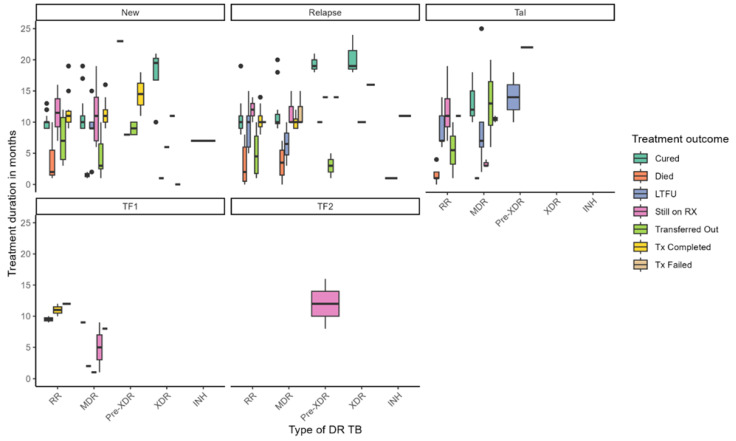
Treatment duration and outcomes by drug-resistant TB type.

Figure 1 illustrates the distribution of treatment duration and outcomes across different types of DR-TB, stratified by TB history—new, relapse, TAL, and treatment failure categories (TF1, TF2). Treatment durations varied widely across DR-TB types, with median durations ranging from approximately 4 to over 20 months. Longer treatment durations were generally associated with favorable outcomes such as cure and treatment completion, while shorter durations were linked to LTFU and mortality. Notably, patients with pre-XDR-TB under the TF2 failure category exhibited the longest treatment durations, exceeding 12 months. Among new TB cases, individuals with MDR-TB and pre-XDR-TB experienced extended treatment courses, whereas RR-TB patients with TF1 failure had shorter durations. Treatment outcomes also varied substantially by resistance type: cure rates were highest among patients with MDR-TB and RR-TB, particularly in new cases. At the same time, mortality was more prevalent in XDR-TB and pre-XDR-TB groups and occurred earlier in treatment. LTFU was observed across all DR-TB categories but was more frequent in MDR-TB and RR-TB cases. Patients still on RX appeared across various subgroups, indicating ongoing clinical challenges, while transferred-out cases were more common among those with longer treatment durations. Treatment failure was primarily concentrated in pre-XDR-TB cases. These findings underscore the complexity of managing DR-TB and highlight the influence of resistance type and treatment history on both duration and outcomes. They point to the need for tailored strategies to reduce mortality, enhance treatment adherence, and support patients throughout prolonged treatment regimens.

### 3.3. Survival Analysis Findings

Survival analysis reveals that the risk of adverse treatment outcomes was most pronounced during the first 6 to 8 months of therapy, with the highest incidence of mortality and LTFU occurring during this period. Conversely, the likelihood of cure and treatment completion peaked between 9 and 12 months. In multivariate Cox regression in Table 3, both primary (HR = 0.39; 95% CI: 0.23–0.68; *p* = 0.0017) and secondary education (HR = 0.50; 95% CI: 0.31–0.85; *p* = 0.0103) were significantly protective. Surprisingly, patients with pre-XDR TB (HR = 0.13; 95% CI: 0.03–0.81; *p* = 0.034) and XDR TB (HR = 0.16; 95% CI: 0.03–0.94; *p* = 0.043) were associated with lower hazard of poor outcomes. Additionally, HIV-negative status was linked with an increased risk of unfavorable outcomes (HR = 1.74; 95% CI: 1.13–2.66; *p* = 0.010). Subgroup analyses further reveal that young adults aged 20–29 experienced disproportionately high LTFU rates, while HIV-positive and underweight individuals had elevated mortality, underscoring the need for targeted interventions within these high-risk groups.

Assessing the potential impact of enhanced clinical governance interventions (e.g., improved patient follow-up, TB/HIV integration, health worker accountability) on treatment outcomes, specifically loss to follow-up, mortality, and treatment completion among DR-TB patients, a predictive analysis approach was employed to evaluate the potential impact of enhanced clinical governance on DR-TB treatment outcomes using proxy indicators derived from the existing dataset. Given the abstract nature of clinical governance, variables such as education level (as a proxy for health communication), previous TB treatment history (reflecting follow-up system gaps), HIV co-infection status (indicating TB/HIV service integration), treatment duration, timing of loss to follow-up, and occupation or income (as indicators of social support) were used to analyze governance-related influences. A logistic regression framework was applied to estimate the likelihood of favorable outcomes—defined as cure or treatment completion—versus unfavorable outcomes, including LTFU, death, treatment failure, or transfer. The analysis incorporated hypothetical scenarios reflecting incremental improvements in clinical governance: a baseline using current data, a moderate intervention scenario (30% reduction in LTFU and 15% increase in adherence among HIV-positive individuals), and a high-impact scenario simulating a 50% reduction in LTFU, full TB/HIV integration, and targeted support for previously treated patients. Predicted probabilities of treatment success were calculated for each scenario, offering insights into the potential gains achievable through strategic system-level reforms.

**Figure 2:**
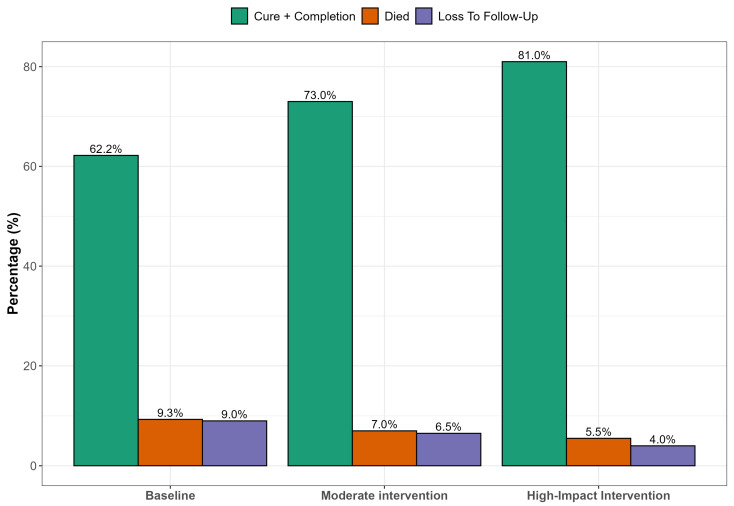
Predicted treatment outcomes under different clinical governance scenarios.

Figure 2 presents simulated results of strengthening clinical governance through targeted interventions that hold substantial promise for improving DR-TB treatment outcomes. Enhanced patient follow-up systems could significantly reduce LTFU, particularly among younger, male patients who are most vulnerable to disengagement from care. Simultaneously, integrating TB and HIV services at the primary care level would likely reduce early mortality by enabling timely co-treatment and improving adherence. Educational interventions such as structured counseling and community outreach are essential for improving outcomes among patients with limited literacy, empowering them to engage more effectively with care. Combined, these strategies within a robust clinical governance framework supported by adherence audits and digital follow-up tools could improve treatment success rates by up to 20 percentage points in high-impact rural settings.

## 4. Discussion

This study underscores critical gaps in DR-TB care that have far-reaching implications for clinical governance and public health systems in rural South Africa. High rates of LTFU and mortality, particularly among HIV-positive individuals, young adults, and those with poor socioeconomic status, highlight systemic weaknesses in adherence support, care continuity, and service integration.

The cohort of patients in this study experienced a cure rate of 36.2% and treatment completion of 26.0%, a 62.2% rate comparable with other settings in Limpopo, South Africa with 57.9% treatment success rate [18] and 62.8% in Zambia [19]. The treatment success rate in this study is lower than those observed in other studies: 69% in Uganda [20], 75.9% in Pakistan [21], and 78.6% in Cameroon [22]. The rate fell short of the 75% target treatment success rate set by the Global End TB strategy of the WHO [15]. Another systematic review that investigated MDR/RR-TB treatment outcomes in Central and West African countries revealed considerable intraregional disparity in MDR-TB treatment outcome, with a treatment success rate of 81% for Central Africa and 69% for West Africa [23].

The higher mortality rates observed in patients with pre-XDR-TB and XDR-TB compared to other TB patients may be attributed to a combination of biological, immunological, and treatment-related factors. These factors contribute to the severity and complexity of the disease, leading to poorer outcomes. XDR-TB characterized by resistance to first-line and second-line drugs complicate treatment alternatives, thereby leading to higher mortality rates [24]. Patients often present with significant comorbid conditions, such as malnutrition (BMI < 18.5 kg/m^2^) and chronic diseases, which exacerbate their health status and increase mortality risk [25]. A substantial proportion of XDR-TB patients are co-infected with HIV, which severely compromises the immune system and contributes to higher mortality rates [26,27]. Previous studies have documented significant association between previous anti-TB treatment (ATT) history and the incidence of pre-XDR-TB and XDR-TB [28,29].

Both primary (*p* = 0.0017) and secondary education (*p* = 0.0103) were significantly protective, suggesting that educational attainment plays a key role in fostering treatment adherence and engagement. The protective effect of basic education suggests that public health interventions must incorporate health literacy components while also addressing structural barriers like transportation, income insecurity, and treatment fatigue [30,31]. Strengthening clinical governance requires systematic follow-up mechanisms, audit-based accountability, and investment in human resources and digital health solutions [32,33,34]. Unexpected findings such as hazard among XDR-TB patients may reflect data limitations or programmatic decisions to transfer patients to higher-level facilities early in treatment. Nevertheless, the use of survival analysis in this context is a powerful tool for identifying critical intervention windows and improving patient tracking in clinical settings, thus providing insights that can inform treatment strategies and improve patient outcomes [35,36,37].

One surprising result of our multivariate analysis was the lower hazard of poor outcome in pre-XDR and XDR-TB patients. Such reverse-sounding outcomes must be due to referral bias hidden below, in which the most severe cases of resistance are first diverted to tertiary centers with improved facilities, thus taken out of the observation period of the present dataset. Residual confounding is also facilitated through the lack of information on treatment regimen, facility, and health system capacity. Pre-XDR and XDR-TB hazard ratios remained of comparable magnitude and sign, indicating that observed relations are unlikely to be artifacts of early censoring, but we see that unmeasured clinical and systemic factors can still affect these findings. The WHO’s Global TB Program defined pre-XDR-TB for the first time in 2021, highlighting the seriousness of these forms of TB. Pre-XDR, defined as TB strains resistant to isoniazid and rifampicin plus resistance to any fluoroquinolone, marks an intermediate but critical stage before XDR-TB, signaling novel treatment challenges. Pre-XDR strains often require individualized regimens lasting 18 months or longer, with reduced success and elevated risk of progression to full XDR status. Pre-XDR-TB points to increasing progression of the severity of the disease, due to resistance to additional medications. Subsequently, a more limited number of options are at a clinician’s disposal to effectively treat it. The survival analysis reveals that being HIV-negative was linked with an increased risk of unfavorable outcomes (HR = 1.74; 95% CI: 1.13–2.66; *p* = 0.010), highlighting a potential gap in support systems for patients not enrolled in HIV care programs. While HIV-positive individuals co-infected with DR-TB often face a higher risk of poor outcomes, paradoxically, HIV-negative DR-TB patients have also been observed to experience unfavorable treatment trajectories in several settings, including South Africa. One possible explanation lies in the supportive care infrastructure that surrounds HIV care, a structure that may not be available to HIV-negative patients. Patients enrolled in HIV care programs benefit from integrated support services that include routine follow-up and clinical monitoring, nutritional supplementation, psychosocial and adherence counseling, social grants (e.g., disability grants linked to HIV), and community-based treatment supporters [38]. These services often overlap with DR-TB care, providing a framework that supports adherence and retention. In contrast, HIV-negative patients may not qualify for many of these programs, leaving them unsupported in navigating a similarly demanding treatment regimen [39].

In Figure 2, we projected treatment outcomes for DR-TB under varying scenarios of clinical governance in a rural South African context. Education was used as a multidimensional proxy, encompassing not only health literacy but also elements of individual empowerment, agency, and capacity to navigate healthcare systems, which are critical components of effective clinical governance in resource-constrained settings. Higher education levels correlate with improved understanding of tuberculosis transmission, better adherence to treatment protocols, and reduced stigma. Previous studies have highlighted that low education is a significant socio-demographic risk factor affecting tuberculosis knowledge and treatment compliance among patients. Educational attainment strongly correlates with health literacy, which influences how individuals understand health information, navigate health services, and make informed decisions [40,41]. Education also enhances a person’s sense of self-efficacy and agency, enabling them to act autonomously in health decision-making and interact assertively with health professionals. The scenarios modeled included a status quo (baseline outcomes under existing clinical governance); moderate improvement (e.g., improved follow-up or partially integrated services); and high-impact scenario (fully integrated TB/HIV services, intensive follow-up, and patient adherence support). At baseline, treatment success remains relatively low (~62.2%), with significant proportions lost to follow-up, mortality, or treatment failure. As governance and integration improve across the modeled scenarios, treatment success rates increase markedly, potentially reaching WHO targets of ≥75% in the high-impact intervention scenario. This projection aligns with findings from several studies demonstrating that strong clinical governance, continuity of care, and integrated TB/HIV management significantly improve DR-TB outcomes. Loveday et al. [42] found that decentralized, patient-centered care improved DR-TB treatment success in KwaZulu-Natal, South Africa, with higher adherence and reduced mortality when clinical teams had dedicated follow-up structures. Kvasnovsky et al. [43] noted that enhanced programmatic supervision and better health worker–patient relationships led to lower default rates in Mozambique and South Africa. The WHO global report [1] highlights treatment success for MDR-TB at 63% globally, but notes that success rates rise in countries employing directly observed therapy, electronic adherence tools, and decentralized services. In contrast, studies in under-resourced settings [44] have documented high default and mortality rates where health systems lacked proper follow-up mechanisms or where integration between TB and HIV services was weak.

The data emphasize that clinical governance is not just an administrative process, it is central to patient outcomes. Strengthening it through integrated service delivery, proactive patient tracing, and resource allocation to rural clinics could significantly reduce the mortality and LTFU burden associated with DR-TB. Moreover, interventions such as community health worker support, digital adherence technologies, and psychosocial counseling can enhance governance indirectly by promoting patient engagement. Clinical governance offers a powerful policy tool, demonstrating that governance enhancements could bridge the gap between current treatment outcomes and global targets. Prioritizing such reforms in high-burden, rural areas like Eastern Cape is essential for reducing the DR-TB epidemic’s toll. Importantly, the study supports the adoption of multi-sectoral strategies that align clinical governance with public health objectives, with a particular focus on high-risk subgroups including youth, individuals with prior TB treatment, and people living with HIV. This combined strategy not only aligns with global TB elimination targets but also fortifies health systems to combat syndemics like TB/HIV, fostering resilience against future health crises.

Despite the contributions of this study, we acknowledge the following limitations that might affect the interpretation and generalizability of the findings and provide directions for future research. First, our current study was based on a retrospective cohort design utilizing secondary data; we acknowledge that the exclusion of patient-level qualitative insights on stigma, transport costs, mental health challenges, and caregiver support is a limitation. Second, treatment adherence stood as a key limitation. The direct measures of adherence (e.g., DOT logs, digital adherence technologies (DATs), or patient-reported measures) were not consistently available in our dataset, limiting our ability to evaluate adherence-related effects. Poor adherence may contribute to LTFU, prolonged treatment duration, and poor outcomes. Third, this study had some missing clinical variables that could be primary predictors of treatment outcome, and their absence from part of the dataset is limiting. It could result in potential underestimation of adverse outcomes like death or LTFU, particularly in those with unrecorded comorbidity or malnutrition. Thus, external validity of the results to all DR-TB patients, especially from resource-poor or poorly recorded settings, is diminished. Improved routine completeness needs to be a focus in future work to facilitate more representative and precise modeling of treatment outcomes for generalizability of outcomes. Fourth, the potential for overestimation and oversimplification is inherent in modeling assumptions, and, therefore, we emphasize the need for future models to incorporate more governance indicators where feasible. Future research should explore prospective or mixed-methods research to better assess adherence behavior, its determinants (e.g., stigma, transport access), and its impact on treatment trajectories.

### Recommendations

Based on the findings of this study, implementable strategies and interventions to enhance the translational value of our study for policymakers and frontline health planners are proposed. First, cost estimates for priority interventions should be in place to guide resource allocation for decentralized adherence support teams (DAS) covering community health worker stipends, transportation, and mobile data, plus the use of digital tools for monitoring and alerts (mHealth) covering implementation of TB-specific DATs such as video-observed therapy (VOT) and integrated TB–HIV community clinics including strengthening integration through minor facility upgrades and task-sharing. Second, digital literacy training for nurses and CHWs should be organized as a structured training module (in collaboration with the National Department of Health eHealth directorate) on patient tracking, data entry, and remote support systems. Peer learning and refresher workshops should be incorporated as quarterly virtual or in-person sessions to ensure continued competency, especially in low-connectivity rural areas. Integration into existing TB registers and DHIS2 should be achieved by ensuring compatibility and minimal duplication of data entry to streamline workflows. Thirdly, community buy-in is vital for improving treatment adherence and stigma reduction. There should be engagement of traditional leaders and faith-based groups to leverage their influence in promoting treatment adherence and destigmatizing DR-TB and TB–HIV co-infection. Community health dialogue is encouraged in the form of monthly community forums using participatory approaches (e.g., health theatre, storytelling) to co-create solutions with patients and families and integration of DR-TB into ward-based outreach teams (WBOTs) by training WBOTs to provide psychosocial support and monitor household TB risks during home visits. Lastly, DR-TB policy should be prioritized in district health plans (DHPs), using evidence from predictive modeling and risk maps.

## 5. Conclusions

This study reveals critical gaps in treatment outcomes for DR-TB patients in rural Eastern Cape, with a treatment success rate of 62.2%—below the WHO target of 75%. Early mortality and LTFU, particularly in the first six to eight months of treatment, underscore the need for intensified support during this vulnerable period. Education level emerged as a significant protective factor, emphasizing the role of health literacy and empowerment in adherence. Unexpectedly, HIV-negative patients experienced poorer outcomes, pointing to a potential gap in support services for non-HIV patients.

These findings provide a foundation for designing responsive, evidence-based health interventions. Health programs should prioritize early patient retention strategies, community-based adherence support, and differentiated models of care that address both clinical and social determinants of DR-TB outcomes. Moreover, strengthening clinical governance—through improved tracking, staff training, and digital integration—can improve program accountability and treatment success. Tailored support for vulnerable groups, including the less educated and HIV-negative patients, should be incorporated into national and district-level DR-TB strategies to achieve equitable outcomes.

## Figures and Tables

**Table 1:** Socio-demographic characteristics of study participants.

Characteristic	N	Overall N = 323	Cured N = 117	Tx Completed N = 84	LTFU N = 29	Tx Failed N = 7	Died N = 30	Transferred Out N = 30	Still on RX N = 26
Age group	323								
0–14		9 (2.8%)	3 (2.6%)	5 (6.0%)	0 (0%)	1 (14%)	0 (0%)	0 (0%)	0 (0%)
15–24		49 (15%)	10 (8.5%)	10 (12%)	6 (21%)	1 (14%)	1 (3.3%)	14 (47%)	7 (27%)
25–44		173 (54%)	68 (58%)	43 (51%)	18 (62%)	5 (71%)	17 (57%)	9 (30%)	13 (50%)
45–64		76 (24%)	29 (25%)	22 (26%)	4 (14%)	0 (0%)	9 (30%)	6 (20%)	6 (23%)
>65		16 (5.0%)	7 (6.0%)	4 (4.8%)	1 (3.4%)	0 (0%)	3 (10%)	1 (3.3%)	0 (0%)
Gender	323								
Male		180 (56%)	59 (50%)	46 (55%)	19 (66%)	3 (43%)	22 (73%)	16 (53%)	15 (58%)
Female		143 (44%)	58 (50%)	38 (45%)	10 (34%)	4 (57%)	8 (27%)	14 (47%)	11 (42%)
Education	323								
No education		64 (20%)	29 (25%)	6 (7.1%)	7 (24%)	1 (14%)	8 (27%)	7 (23%)	6 (23%)
Primary		76 (24%)	24 (21%)	29 (35%)	3 (10%)	4 (57%)	10 (33%)	4 (13%)	2 (7.7%)
Secondary		150 (46%)	50 (43%)	35 (42%)	17 (59%)	2 (29%)	11 (37%)	17 (57%)	18 (69%)
Tertiary		33 (10%)	14 (12%)	14 (17%)	2 (6.9%)	0 (0%)	1 (3.3%)	2 (6.7%)	0 (0%)

**Table 2:** Clinical characteristics of study participants.

Characteristic	N	Overall N = 323	Cured N = 117	Tx Completed N = 84	LTFU N = 29	Tx Failed N = 7	Died N = 30	Transferred Out N = 30	Still on RX N = 26
BMI category	323								
Under-weight		86 (27%)	24 (21%)	21 (25%)	8 (28%)	3 (43%)	8 (27%)	10 (33%)	12 (46%)
Normal weight		152 (47%)	59 (50%)	39 (46%)	14 (48%)	3 (43%)	17 (57%)	11 (37%)	9 (35%)
Over-weight		51 (16%)	19 (16%)	11 (13%)	5 (17%)	1 (14%)	5 (17%)	7 (23%)	3 (12%)
Obese		34 (11%)	15 (13%)	13 (15%)	2 (6.9%)	0 (0%)	0 (0%)	2 (6.7%)	2 (7.7%)
Patient category	323								
New		169 (52%)	69 (59%)	55 (65%)	6 (21%)	0 (0%)	10 (33%)	20 (67%)	9 (35%)
Relapse		103 (32%)	42 (36%)	23 (27%)	8 (28%)	5 (71%)	13 (43%)	6 (20%)	6 (23%)
Tal		38 (12%)	3 (2.6%)	4 (4.8%)	14 (48%)	0 (0%)	6 (20%)	4 (13%)	7 (27%)
TF1		11 (3.4%)	3 (2.6%)	2 (2.4%)	1 (3.4%)	2 (29%)	1 (3.3%)	0 (0%)	2 (7.7%)
TF2		2 (0.6%)	0 (0%)	0 (0%)	0 (0%)	0 (0%)	0 (0%)	0 (0%)	2 (7.7%)
Previous Drug History	323								
New		166 (51%)	68 (58%)	53 (63%)	6 (21%)	0 (0%)	10 (33%)	20 (67%)	9 (35%)
PT1		125 (39%)	48 (41%)	28 (33%)	10 (34%)	6 (86%)	15 (50%)	8 (27%)	10 (38%)
PT2		31 (9.6%)	1 (0.9%)	3 (3.6%)	12 (41%)	1 (14%)	5 (17%)	2 (6.7%)	7 (27%)
UNK		1 (0.3%)	0 (0%)	0 (0%)	1 (3.4%)	0 (0%)	0 (0%)	0 (0%)	0 (0%)
HIV status	323								
Positive		200 (62%)	65 (56%)	50 (60%)	18 (62%)	6 (86%)	26 (87%)	17 (57%)	18 (69%)
Negative		123 (38%)	52 (44%)	34 (40%)	11 (38%)	1 (14%)	4 (13%)	13 (43%)	8 (31%)
Comorbidities	323								
None		271 (84%)	100 (85%)	69 (82%)	27 (93%)	6 (86%)	24 (80%)	22 (73%)	23 (88%)
HTN		13 (4.0%)	5 (4.3%)	6 (7.1%)	0 (0%)	0 (0%)	1 (3.3%)	1 (3.3%)	0 (0%)
HTN and T2DM		2 (0.6%)	0 (0%)	0 (0%)	0 (0%)	0 (0%)	0 (0%)	2 (6.7%)	0 (0%)
T2DM		5 (1.5%)	1 (0.9%)	1 (1.2%)	0 (0%)	0 (0%)	1 (3.3%)	1 (3.3%)	1 (3.8%)
T2DM and mental illness		1 (0.3%)	0 (0%)	1 (1.2%)	0 (0%)	0 (0%)	0 (0%)	0 (0%)	0 (0%)
Epilepsy		5 (1.5%)	3 (2.6%)	1 (1.2%)	0 (0%)	0 (0%)	1 (3.3%)	0 (0%)	0 (0%)
Mental illness		2 (0.6%)	1 (0.9%)	0 (0%)	0 (0%)	0 (0%)	0 (0%)	1 (3.3%)	0 (0%)
Hearing loss		22 (6.8%)	7 (6.0%)	5 (6.0%)	2 (6.9%)	1 (14%)	3 (10%)	3 (10%)	1 (3.8%)
Allergies		1 (0.3%)	0 (0%)	1 (1.2%)	0 (0%)	0 (0%)	0 (0%)	0 (0%)	0 (0%)
HTN and Allergies		1 (0.3%)	0 (0%)	0 (0%)	0 (0%)	0 (0%)	0 (0%)	0 (0%)	1 (3.8%)
Type of TB	323								
PTB		319 (99%)	116 (99%)	83 (99%)	29 (100%)	7 (100%)	29 (97%)	30 (100%)	25 (96%)
EPTB		4 (1.2%)	1 (0.9%)	1 (1.2%)	0 (0%)	0 (0%)	1 (3.3%)	0 (0%)	1 (3.8%)
Type of resistance	323								
Mono		144 (45%)	51 (44%)	42 (50%)	10 (34%)	2 (29%)	20 (67%)	11 (37%)	8 (31%)
Poly		179 (55%)	66 (56%)	42 (50%)	19 (66%)	5 (71%)	10 (33%)	19 (63%)	18 (69%)
Type of DR-TB	323								
RR		145 (45%)	51 (44%)	43 (51%)	10 (34%)	2 (29%)	19 (63%)	12 (40%)	8 (31%)
MDR		139 (43%)	54 (46%)	36 (43%)	15 (52%)	4 (57%)	8 (27%)	9 (30%)	13 (50%)
Pre-XDR		21 (6.5%)	4 (3.4%)	3 (3.6%)	2 (6.9%)	1 (14%)	1 (3.3%)	6 (20%)	4 (15%)
XDR		15 (4.6%)	7 (6.0%)	1 (1.2%)	2 (6.9%)	0 (0%)	1 (3.3%)	3 (10%)	1 (3.8%)
INH-R		3 (0.9%)	1 (0.9%)	1 (1.2%)	0 (0%)	0 (0%)	1 (3.3%)	0 (0%)	0 (0%)
Treatment duration	323	10 (9, 11)	10 (9, 12)	11 (10, 12)	9 (6, 10)	10 (10, 14)	2 (1, 5)	4.5 (2, 10)	10 (7, 14)

Tal—treatment after LTFU; TF1—treatment failure with first-line drug; TF2—treatment failure with second-line drug; PT1—previously treated with first-line drugs; PT2—previously treated with second-line drugs; UNK—unknown; HTN—hypertension; T2DM—type 2 diabetes mellitus; PTB—pulmonary tuberculosis; EPTB—extrapulmonary tuberculosis; n (%); median (Q1, Q3); RR—rifampicin-resistant; MDR—multidrug-resistant; XDR—extensively drug-resistant; INH-R—isoniazid-resistant.

**Table 3:** Cox proportional hazards model result.

Variable	Hazard Ratio (HR)	*p*-Value	Hazard Ratio (HR)	*p*-Value
Education				
Primary	0.49760	0.00963 **	0.39300	0.00171 **
Secondary	0.56050	0.01176 *	0.50400	0.01027 *
Tertiary	0.87770	0.68792	1.08400	0.82279
Income				
Self-employed	0.52230	0.51800	0.35600	0.31491
Casual	3.79500	0.18600	4.89200	0.12986
Salary	0.77390	0.46300	0.58500	0.18758
UIF	0.00000	0.99400	0.00000	0.99850
DG	1.55600	0.23000	1.75000	0.26117
BMI category				
Normal weight	1.12950	0.59800	1.09000	0.75338
Over-weight	1.44820	0.21500	1.46100	0.25666
Obese	1.49590	0.21700	1.23600	0.56530
Previous drug history				
PT1	0.87540	0.47334	0.60600	0.62970
PT2	0.05110	0.00319 **	0.00000	0.99303
Comorbidities				
HTN	0.96600	0.94100	0.76700	0.61401
HTN and T2DM	0.00000	0.99700	0.00000	0.99910
T2DM	0.89500	0.91300	1.60900	0.65469
Epilepsy	2.02200	0.99700	1.99300	0.26872
Mental illness	1.19400	0.16900	Inf	0.99320
Hearing loss	0.71560	0.86000	0.63100	0.27288
Patient category				
Relapse	0.84490	0.38301	1.95300	0.52059
TAL	0.16650	0.00248 **	1.79700	0.63824
TF1	1.17100	0.78951	3.48600	0.29504
TF2	0.00000	0.99502	9.50500	0.99984
Type of resistance				
Polyresistance	0.70660	0.0623	1.14600	0.85914
Type of DR-TB				
MDR	0.85530	0.41557	0.79100	0.75999
Pre-XDR	0.22780	0.00528 **	0.13400	0.03446 *
XDR	0.50600	0.10594	0.16400	0.04264 *
INH	1.7849	0.56627	2.33600	0.41766
HIV status				
Negative	1.31670	0.13000	1.73500	0.01038 *

UIF—unemployment insurance fund, DG—disability grant, PT1—previously treated with first-line drugs; PT2—previously treated with second-line drugs; HTN—hypertension; T2DM—type 2 diabetes mellitus; TAL—treatment after LTFU; TF1—treatment failure with first-line drugs; TF2—treatment failure with second-line drugs; * significant; ** highly significant.

## Data Availability

Data can be requested from the corresponding author upon reasonable request.

## Abbreviations

DR-TB	Drug-resistant tuberculosis
LTFU	Loss to follow up
IQR	Inter quartile range
HR	Hazard ratio
MDR-TB	Multidrug-resistant tuberculosis
XDR-TB	Extensively drug-resistant tuberculosis
HIV	Human immunodeficiency virus
BMI	Body mass index
WHO	World Health Organization
Tal	Treatment after loss to follow up
TF1	Treatment failure after first-line drug
TF2	Treatment failure after second-line drug
PT1	Previously treated with first line-drug
PT2	Previously treated with second-line drug
UNK	Unknown
HTN	Hypertension
T2DM	Type 2 diabetes mellitus
PTB	Pulmonary tuberculosis
EPTB	Extrapulmonary tuberculosis
TB	Tuberculosis
RR-TB	Rifampicin-resistant tuberculosis
INH-R	Isoniazid-resistant
CI	Confidence interval
UIF	Unemployment insurance fund
DG	Disability grant
ATT	Anti-tuberculosis treatment
DHPs	District health plans
DAS	Decentralized adherence support teams
DATs	Digital adherence technologies
VOT	Video-observed therapy
CHW	Community health workers
DHIS2	District Health Information Software
WBOTs	Ward-based outreach teams
